# Perceptions of substance use, treatment options and training needs among Iranian primary care physicians

**DOI:** 10.1186/1475-9276-4-7

**Published:** 2005-06-15

**Authors:** Anthony Shakeshaft, Bijan Nassirimanesh, Carolyn Day, Kate A Dolan

**Affiliations:** 1 Program of International Research and Training (PIRT), National Drug and Alcohol Research Centre (NDARC), University of New South Wales, Sydney, Australia; 2 3^rd ^Floor, Building 19, Soori Alley, Moradi Alley, Bani-Hashemi St, Resalat Boulevard, Tehran, Iran; 3 National Health and Medical Research Centre Fellow, The National Centre in HIV Epidemiology and Clinical Research, University of New South Wales, Sydney, Australia

## Abstract

In order to be optimally effective, continuing training programmes for health-care professionals need to be tailored so that they target specific knowledge deficits, both in terms of topic content and appropriate intervention strategies. A first step in designing tailored treatment programmes is to identify the characteristics of the relevant health-care professional group, their current levels of content and treatment knowledge, the estimated prevalence of drug and alcohol problems among their patients and their preferred options for receiving continuing education and training. This study reports the results of a survey of 53 primary care physicians working in Iran. The majority were male, had a mean age of 44 years and saw approximately 94 patients per week. In terms of their patients' drug use, primary care physicians thought most patients with a substance use problem were male, women were most likely to use tobacco (52%), opium (32%) and marijuana/hashish and young people were most likely to use tobacco, alcohol, marijuana and heroin. Counselling and nicotine patches were the treatments most commonly provided. Although the majority (55%) reported referring patients to other services, more than a third did not. Most primary care physicians reported being interested in attending further training on substance abuse issues. The implications of these data for ongoing education and training of primary care physicians in Iran are discussed.

## Background

The collection and interpretation of accurate descriptive data is an essential step in ultimately reducing the burden of harm associated with substance use [1,2]. Traditionally, these data have been sought to identify the characteristics of at-risk individuals or groups, quantify the size of the problem (incidence and prevalence) and, when repeated on the same population and using the same measures, monitor trends over time and assess the effects of interventions. More recently, these same principles have been applied to health care providers, as a mechanism for improving their quality of care [3]. As for at-risk individuals, accurate descriptive data identify the characteristics of health care providers and their knowledge gaps, and facilitate quality assurance monitoring of their practice over time.

Descriptive data also help optimise the cost-effectiveness of interventions in two ways. Firstly, at an individual level, they enable greater accuracy in tailoring interventions and treatment goals to specific problem behaviours [4]. Secondly, at a systems level, they provide an empirical rationale for the equitable distribution of intervention resources, a notion espoused by the World Health Organisation (WHO) as a key principle of accessibility to primary health care, in which core services available to all are complemented by interventions targeting those at increased risk [5].

As is typically the case internationally, the centrality of primary care in Iran is reflected in its role as the usual first point of contact with the formal health-care system. It is the principal juncture at which a range of health services can be mobilised and co-ordinated in order to manage complex health problems, such as substance abuse.

In assessing the potential to improve the quality and equity of primary health care for substance abuse problems in Iran, an initial step is to determine the characteristics of primary care practitioners, including their knowledge and perceptions of substance use, as well as identify their requirements for on-going education and training.

Therefore, this study has three aims. Firstly, to describe the demographic and practice characteristics of a sample of General Practitioners (GPs) working in Iran. Secondly, to identify their perceptions of substance use among their patients and their current practice regarding treatment. Thirdly, to identify Iranian GPs' interest in on-going education and training, their knowledge gaps and their preferences for the format of such training.

## Method

### Sample

GPs attending one of two mandatory training workshops in Tehran, Iran, were asked to complete a written questionnaire. Both workshops were held in the winter of 2003 and were attended by 50 GPs each. The topic of one workshop was an examination of psychiatry and medicine and the other focused on surgery. GPs throughout Iran travel to Tehran to attend these workshops, because they require ongoing accreditation to renew their licence to practice.

### Measures

The pen-and-paper questionnaire was devised in English and translated into Farsi. Specific questions were asked in a number of domains, covering primary care practitioners' demographic and practice characteristics, and their perceptions of: substance use among their patients; substance use treatment characteristics; their current practice in regard to treatment for substance use; and their on-going training and education preferences. The full survey is available at the PIRT website .

### Procedure

The questionnaire items were devised in conjunction with an Iranian GP (BN), who also distributed the questionnaires at the GP workshops. The completed questionnaires were photocopied and couriered to the research centre in Sydney, Australia. GPs' responses were translated back from Farsi to English, entered into an Excel spreadsheet and transferred to SPSS for Windows, Version 12, for analyses. Descriptive statistics report means, percentages and ranges as appropriate.

## Results

### Sample

Of the 100 GPs who attended one of the workshops, 53 completed the questionnaire.

### GP demographic and practice characteristics

The majority of GPs were male (84%) with a mean age of 44 years (range 26 to 74 years). Their year of graduation from medical school ranged from 1950 to 2001 and the mean number of years practicing as a primary care physician was 16. The estimated mean number of patients seen per week was 94, of whom an estimated mean of 12 were substance users. Thirty eight percent of GPs reported seeing more than five substance using patients per week, while 2% said they have no substance using patients. GPs visited an estimated mean of 15 substance users per week, as opposed to seeing them in their primary care practice.

### GPs' perceptions of substance use characteristics of their patients

Table 1 reports GPs' perceptions of substance use characteristics among their patients.

**Table 1:** GPs' perceptions of substance use characteristics of their patients (N = 53)

***Characteristic***^*a*^	***% or n***
*Of your patients who have drug problems, what % are:*	
Male	81%
	
*What is the cause of addiction?*^*b*^	
Emotional	49%
Social	45%
Family	35%
Economic	31%
Leisure	31%
Wrong role models	26%
	
*In the last 5 years, have drug addiction trends increased, decreased, unchanged:*	
Increased	94%
	
*In the last 5 years, drug abuse increased more for males or females*^*c*^*:*	
Females	42%
Males	38%
Same	18%
	
*If increased, why?*^*d*^	
Social/family issues	22
Economic reasons	20
Unemployment	17
Easy access to drugs	17
Lack of recreation/sport facilities	10
Youth-related issues	9
Ineffective law enforcement/government action	6
Inadequate education	2
Other	10
	
*What is your view of drug addicts?*^*b*^	
Psychologically ill	82%
Physically ill	22%
Burden on society	22%
Criminals	2%
	
*Level of awareness about substance abuse*^*e*^	
Moderate	54%
High	27%

^a^Data missing: n = 2–5

^b^Does not sum to 100%, because some GPs gave more than one answer and some gave no answer

^c^Don't know = 2%

^d^Does not sum to 53, because some GPs gave more than one answer and same gave no answer

^e^Awareness rated on Likert scale from 1 (very unaware) – 10 (very aware): low = 1–3; moderate = 4–8; high = 8–10

Most GPs viewed substance use problems as a psychological issue (82%) and that most patients with a substance use problem are male (81%). A substantial percentage of GPs reported moderate (54%) or high (27%) awareness of substance abuse issues. Some GPs thought emotional (49%) or social (45%) factors were the cause of addiction. Almost all GPs (94%) thought drug addiction trends in Iran have increased in the previous five years and a comparable percentage thought the increase had been greater for females (42%) compared to males (38%). The most commonly cited reasons for increased drug addiction were social or family issues (n = 22), followed by economic factors (n = 20).

### GPs' perceptions of substance use characteristics by sex and age

The majority of GPs perceived that women were most likely to use tobacco (52%), opium (32%), marijuana/hashish (32%) and heroin (14%). The majority of GPs thought young people (aged less than 25 years) were willing to use tobacco (97%), alcohol (63%), marijuana (56%) and heroin (53%), but were relatively unlikely to use black water (the dissolved residue from an opium pipe) (7%) and barbiturates (7%). The majority of GPs thought people aged 25 to 44 years were willing to use opium (84%), alcohol (61%) and heroin (52%), while those aged at least 45 years, were most willing to use opium (60%), followed by black water (28%) and alcohol (22%).

### GPs' perceptions of patient and treatment characteristics associated with interventions for substance use

Table 2 reports GPs' perceptions of patient and treatment characteristics associated with interventions for substance use.

**Table 2:** GPs' perceptions of patient and treatment characteristics associated with interventions for substance use (N = 53)

***Characteristic***^*a*^	***% or n***
*Who is more willing to quit?*	
Males	62%
Don't know	11%
	
*Target groups for campaigns against abuse?*	
Youth ≤ 25 years	68%
Urban	5%
Users and drug and alcohol organisations	8%
Jails	3%
Other	16%
	
*What is the most important motive for quitting?*^*b*^	
Family or marital pressure	60%
Economic pressure	44%
Tired of addiction	42%
Legal issues	22%
Employment issues	12%
Physical illness	4%
Other	4%
	
*What is a suitable substitute for substance use?*^*c*^	
Drug or other treatment	18
Societal, family or community factors	16
Recreational/sporting activities	11
Employment	10
Restricted access or punitive (eg. jail)	3
Nothing or don't know	7
	
*Most effective methods for quitting nicotine?*^*c*^	
Counselling or psycho-social interventions	22
Recreational or sporting activities	9
Pharmacotherapy	7
Combination of therapies	3
Alternative therapies	1
Individual's determination to quit	2
Don't know	3
	
*Important side-effects of quitting nicotine?*^*c*^	
Restlessness/irritability	18
Physical symptoms/effects	13
Emotional symptoms/effects	7
Non-specified withdrawal symptoms	4
None	7
	
*Effectiveness of complementary medicines for nicotine dependence?*^*c*^	
Not effective/minimal	10
Somewhat effective	12
Effective	6
Other	6
Don't know	12
	
*Suitability of GP to provide drug services*	
Suitable	71%

^a^Data missing: n = 3–12

^b^Does not sum to 100%, because these questions asked separately

^c^Does not sum to 53, because some GPs gave more than one answer and some gave no answer

The majority of GPs thought males were more willing to quit substance use (62%) and that those aged 25 years or less were the most appropriate group at which to target campaigns against substance misuse (68%). The majority of GPs thought family or marital pressure was the main motive for quitting substance use (60%), followed by economic pressure (44%), being tired of addiction (42%) and legal problems (22%). The most commonly cited suitable substitute for drug use was some type of treatment (n = 18). With regard to nicotine, the most commonly cited effective method for quitting use was counselling or psycho-social intervention (n = 22), while the most commonly cited important side-effect was restlessness or irritability (n = 18) and relatively few (n = 6) regarded complementary approaches, such as hypnotherapy and chiropractic therapy, as effective. The majority of GPs (71%) thought primary care was a suitable setting for delivery of services to patients with substance use problems.

### GPs' current practice in regard to treatment for substance misuse

Table 3 reports GPs' current practice.

**Table 3:** GPs' current practice in regard to treatment for substance misuse (N = 53)

***Current treatment practices***^*a*^	***% or n***
*I conduct diagnostic physiological assessments*	
No	75%
Blood, heart, lung test	18%
Blood test only	4%
Spirometry	4%
	
*I prescribe medications for detoxification*	
No	61%
	
*Treatments I commonly use are*^*b*^*:*	
Counselling or psychotherapy	20
Nicotine patch	11
Non-specified medication	8
Personal/family responsibility	4
Alternative therapy	3
Other	7
Don't know	4
	
*I provide the following relapse information*^*b*^*:*	
Avoid or reduce contact with drug users	8
Psychological/personal determination factors most important	7
Family/friends/social support important	6
Recommend counselling or pharmacotherapy	6
No relapse problems	2
Don't know	2
Other	10
	
*I refer to social workers or detoxification units*	
No	39%
Yes, some patients	43%
Yes, all patients	7%
Yes, if patient is willing	5%
Not applicable	7%

^a^Data missing: n = 7–25

^b^Does not sum to 53, because some GPs gave more than one answer and some gave no answer

The majority of GPs reported that they did not conduct diagnostic physiological assessments (75%) and that they did not prescribe medications for detoxification (61%). The most commonly provided treatments reported were counselling or psychotherapy (n = 20), followed by nicotine patches (n = 11). The most commonly provided relapse information was to avoid or reduce contact with other drug users (n = 8). The majority of GPs said that they referred patients to a social worker or detoxification unit (55%), while a substantial percentage reported not referring at all (39%).

### GPs' on-going training and education preferences

The majority of GPs reported that they were interested in attending seminars and further training related to substance abuse issues (89%) and the most preferred format for such training was two days over a two week period (54%), followed by two days in one week (25%) and two consecutive days (13%). Six per cent reported no preference.

## Discussion

The potential to improve the quality and equity of primary health care for substance abuse problems in Iran, is clearly evidenced by the low proportion of GPs (27%) who currently rate their level of awareness of substance abuse issues as high, despite having been practicing as GPs for a mean of 16 years. This apparent lack of knowledge and skills regarding substance abuse issues is consistent with views of GPs in Western countries [6,7]. The appropriateness of on-going education and training workshops as a mechanism to realise this potential for improvement is reflected by the high proportion of GPs (89%) who indicated an interest in attending such seminars.

The results of this survey also identify potential topics for these workshops that are most likely to maximise their impact. That a minority of GPs (22%) consider patients with substance use problems as being physically ill (Table 1) and do not regard physical illness as an important motive for quitting substance use (4%), suggests a teaching focus on the development of physiological dependence following initiation to drug use would improve GPs' understanding of the nature and impact of physiological dependence. In turn, this may encourage the high proportion of GPs who do not currently prescribe medication for detoxification (61%) or conduct physiological assessments (75%), to review their practice (Table 3).

Similarly, a greater number of GPs may be more inclined to discuss problems associated with relapse to drug use, complemented by monitored pharmacotherapy, or on-going counselling (Table 3). Although a lack of resources may help explain a reluctance to prescribe medication and conduct physiological assessments, GP training could emphasise the need to address issues around physical dependence, as well as the social/familial/economic impacts, along with basic physiological tests that could be conducted periodically. GPs would likely find this approach acceptable, given some cited drug substitution as a suitable treatment and reported currently prescribing nicotine patches (Table 3). Such a change in Iranian GPs' practice would also appropriately reflect the apparent improved rates of quitting smoking, compared to monotherapy or unassisted attempts to quit, in the international literature [8-11].

GPs are clearly aware of the importance of prevention, as evidenced by two-thirds thinking that campaigns against drug abuse would most effectively target young people, presumably prior to the onset of significant dependence. This suggests GPs would be prepared to become involved in preventive activities, in addition to their more traditional role of providing treatment. Therefore, training programmes might also usefully provide GPs with resources and ideas that they can implement to facilitate effective prevention. For example, cost-effective GP prevention efforts would target defined groups with high prevalence rates of use for particular substances: young people aged less than 25 years could be targeted with programs aimed at minimising harm associated with smoking, alcohol and marijuana use, given the majority of GPs perceive that young people are relatively willing to use tobacco (97%), alcohol (63%) and marijuana or hashish (56%). A particular area of concern, given disproportionately greater probability of substantial negative social and health outcomes, is that 53% of GPs perceive young people are willing to use heroin.

Another example may be the apparent importance of targeting womens' use of tobacco, opium and marijuana or hashish, given 52%, 32% and 32% of GPs' respectively, perceive that these are the substances most likely to be used by women. As such, GPs' consultations with their female patients could emphasise the importance of not smoking any substances to reduce the possibility of deleterious health outcomes.

In addition to the potential to improve the quality and equity of primary health care for substance abuse problems in Iran at the GP level, there also appear to be opportunities at the administrative level. For example, there may be value in the Health Department exploring opportunities for formalising referral networks, in order to achieve clarity around pathways from primary care settings to more specialist clinical settings, given only about one-third of GPs (39%) report referring to social workers or detoxification units. There may also be scope to make pharmacotherapies of demonstrated cost-effectiveness more readily available to GPs to prescribe, along with greater access to relevant physiological tests. Clearly, the feasibility of these options needs to be considered within the context of the Iranian health care system.

The generalisability of these results to the population of primary care practitioners in Iran is likely to be limited by two main factors. Firstly, only a small proportion of GPs in Iran participated in this study. However, an attempt was made to minimise this limitation by ensuring that the workshops from which these practitioners were recruited was unrelated to substance use and, as such, the views of these practitioners are likely to represent typical levels of interest and knowledge in drug- and alcohol-related issues. The likelihood that these GPs are typical of Iranian GPs is further indicated by the mandatory nature of the workshops: that they are compulsory for ongoing professional accreditation in Iran decreases the possibility that workshops are only attended by a self-selected sub-group of GPs.

Secondly, since only 53% of those who attended a workshop completed a questionnaire, it may be that those with an interest in the drug and alcohol field, or those who see a relatively large proportion of patients with substance misuse problems, were more likely to complete the survey. The implication of that scenario is that these results are likely to represent a bias towards GPs with greater knowledge or experience in providing health care services to patients with drug and alcohol problems, in which case identified knowledge deficits and training needs would actually be greater among the general population of GPs in Iran.

Future international collaborative research efforts could be improved in a number of ways. Firstly, the process of translation and back translation is susceptible to error and is difficult to monitor accurately. A possible method of reducing translation errors could be to employ translators with substantive knowledge in the drug and alcohol field. Secondly, working with one local researcher to apply a standardised and agreed protocol for distributing the survey, would minimise the possibility of bias resulting from different people administering the survey in different ways. Thirdly, clarity regarding definitions of key terms during the development of survey items and protocols is crucial. Using the example of this study, terms such as 'drug addict' and 'complementary medicine' should be clearly defined.

## Competing interests

The author(s) declare that they have no competing interests.
